# The landscape of treatment of chronic kidney disease in hereditary ATTR amyloidosis

**DOI:** 10.1186/1750-1172-10-S1-I12

**Published:** 2015-11-02

**Authors:** Luisa Lobato

**Affiliations:** 1 Department of Nephrology and Unidade Corino de Andrade, Hospital de Santo António, Centro Hospitalar do Porto - CHP, 4099-001 Porto, Portugal; 2 Multidisciplinary Unit for Biomedical Research - UMIB, Instituto de Ciências Biomédicas Abel Salazar, University of Porto, 4050-313 Porto, Portugal

## 

Hereditary transthyretin amyloidosis (ATTR) is an autosomal dominant disease, caused by mutations in the *TTR* gene, the most prevalent being V30M. Although each TTR variant has a different involvement, peripheral neuropathy and cardiomyopathy are predominant. Kidney deposits are well recognized since the original description of the disease as well as a renal phenotype is also likely. Kidney involvement with proteinuria and progressive renal failure is a major cause of morbidity and mortality in ATTR amyloidosis. However, kidney impairment is not as common as in AA or AL amyloidosis. Epidemiological studies from Portugal and Sweden reveal that approximately one third of the patients display varying degrees of albuminuria and renal dysfunction. Dialysis improves the prognosis and survival, but morbidity and mortality are higher than in other populations given this treatment.

The low level of proteinuria or slight renal impairment does not suppose a so heavy glomerular and vascular deposition of amyloid as in ATTR, particularly in V30M mutation. Moreover, severity of renal amyloid deposition did not consistently parallel that of myelinated nerve fiber loss, with dissociation between kidney and neurological involvement. These are pitfalls that motive troublesome criteria for therapy in ATTR nephropathy. Twenty five years ago, liver transplantation (LT) was introduced as a treatment of ATTR, since this suppresses the production of circulating mutant TTR and theoretically stops the amyloid formation and disease progression, but uncertainties remain about its role on kidney deposits. Renal dysfunction pre LT and acute renal injury post LT are risk factors for chronic renal disease development after LT.

The approach for stage 4 or 5 kidney disease remains the combined or sequential liver-kidney transplantation in eligible patients. However, in the majority of patients, hemodialysis is the unique option even in the presence of a well-functioning liver graft. Tafamidis, a TTR stabilizing drug, was described as having a benefit effect on albuminuria and renal function. Non-steroidal anti-inflammatory drugs showed efficacy stabilizing ATTR in early forms of amyloid neuropathy. However, studies with diflunisal noted decline in eGFR even with exclusion of patients with significant renal impairment.

RNA interference (RNAi) is an endogenous cellular mechanism for controlling gene expression with application to ATTR neuropathy. The therapeutic value in kidney disease with successfully silenced intraglomerular genes in mouse models was proved, raising the possibility of a future role for renal ATTR amyloidosis. Antisense oligonucleotides are also under clinical trials; renal epithelial cells efficiently take up oligonucleotides without apparent degradation, rendering the kidney an excellent target for site-directed antisense therapy, but also a site of antisense toxicity. Other several natural products that inhibit TTR amyloid fibril formation where progressively investigated to stop neuropathy, but the specificity for renal disease was never evaluated. Until now, doubts remain about the role of new therapies in nephropathy, if there are preferential indications for a specific one and if dialysis patients should be included in a particular treatment.

